# Future prospects of pediatric lung transplantation

**DOI:** 10.1016/j.jhlto.2026.100606

**Published:** 2026-06-10

**Authors:** Ernestina Melicoff, David Moreno McNeill, Christian Benden

**Affiliations:** a Baylor College of Medicine and Texas Children’s Hospital, Houston, TX; b Boston Children’s Hospital and Harvard Medical School, Boston MA; c University of Zurich Medical Faculty, Zurich, Switzerland

**Keywords:** pediatric, lung transplant, children, donor, frailty, outcomes, allograft

## Abstract

This special issue of the Journal of Heart Transplantation Open is dedicated to Pediatric Thoracic Transplantation. The authors will discuss subjects related to pediatric lung transplantation such as access to donor organs, allograft dysfunction and role of functional status in outcomes.

## Pediatric lung transplantation: future direction of the field

**Figure 1:**
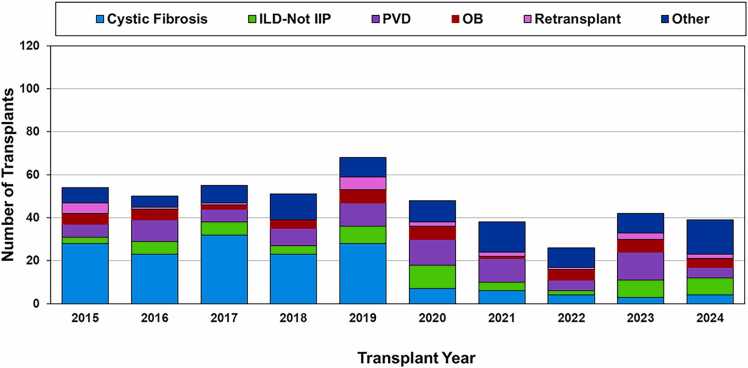
Modified from The International Thoracic Organ Transplant (TTX) Registry of the International Society for Heart and Lung Transplantation 2026 Annual Report. Annual Number of Pediatric Lung Transplants by Diagnosis 2015 – 2024.^3^

Pediatric lung transplantation is a well-established therapeutic modality for a select group of children with severe lung disease.^1^ This intervention offers a survival advantage and enhances the quality of life for patients who lack alternative treatment options. Historically, cystic fibrosis (CF) was a prevalent indication for lung transplantation. In the United States the United Network for Organ Sharing (UNOS) has reported a total of 1511 pediatric lung transplants between 1988 and 2024, 46.8% were due to CF and as per the latest report published by the International Society for Heart and Lung Transplantation (ISHLT) 50% of transplant recipients in Europe had CF.^2^, ^3^ The change in indications for transplant in children can be seen in Figure 1.

The introduction of cystic fibrosis transmembrane conductance regulator (CFTR) modulator therapy has transformed the landscape of cystic fibrosis and pediatric lung transplantation, resulting in a consistent decline in the proportion of lung transplantation performed for end-stage CF.^4^ Notably, following the approval of elexacaftor/tezacaftor/ivacaftor in 2019 the number of CF-related pediatric lung transplants has reached an all-time low, as evidence by only 19 children undergoing lung transplantation for CF in the United States between 2020 and 2024.^2^ The introduction of CF modulator therapy has revolutionized the management of cystic fibrosis and pediatric lung transplantation.

With the declining number of lung transplants for CF, there has been a steady increase in the proportion of pediatric lung transplants for other indications. The main diagnosis for transplant in children is now pulmonary vascular disease (PVD) and childhood interstitial lung disease (chILD). Avidimiretz, et al., published the steady increase in chILD as an indication for pediatric lung transplant from less than 5% of the transplants in 1995 to over 20% in 2025. Similarly, PVD as the indication for lung transplant has increased from 10% in 1995 to 15% in 2025.^5^

While the total volume of pediatric lung transplants is declining, there has been a notable shift in indications and an increase in patient acuity. The use of extracorporeal membrane oxygenation (ECMO) as a bridge to transplant has steadily increased; notably, no patients were bridged with ECMO in 2004, compared to 16.7% in 2018.^6^ Despite the increased reliance on this support, post-transplant survival rates remain comparable. However, the rising complexity of these candidates necessitates continued monitoring of clinical outcomes.^7^

There is a gap in the literature regarding the use of ECMO as a bridge to pediatric lung transplantation in an international setting. Most of the data from outside of the United States is limited to small case series or case reports. Highlighting the need for a robust international registry to help the lung transplant community learn and support this small but important field.

## Access to pediatric organs

Even though the demand for pediatric lung transplants has decreased in recent years, it is still a valuable therapy for selected children with lung disease. We know lungs are a scarce resource, and children’s smaller chest cavities represent a higher challenge to find appropriate organs. Keeshan et al^8^ reported pediatric patients had higher mortality on the lung wait list when compared to adults (22% vs 14.4%). Expanding access to pediatric organs is paramount to provide this life-saving therapy for children.

There have been different strategies to expand access to organs appropriate for pediatric patients. These include living and deceased lobar transplantation, transplanting ABO incompatible organs, donation after cardiac death (DCD), and using ex-vivo lung perfusion (EVLP) to optimize marginal organs previously not considered for transplantation.^9^, ^10^, ^11^ All these strategies are promising to expand the donor pool for pediatric patients.

Lung allocation plays a critical role in access to organs. The Organ Procurement and Transplantation Network (OPTN) in the United States manages the allocation system for organ transplantation. The lung transplant allocation system has undergone 4 major eras. When created in 1990 the system had no pediatric distinction and was based on ABO blood type, time on the waitlist and geographic proximity. In 2005 the Lung Allocation Score (LAS) was implemented. The score was based on a composite of waitlist mortality and post-transplant survival for candidates 12 years or older; children 0–11 continued being allocated based on priority system. The LAS did have a positive impact on waitlist mortality.^11^ In 2017 the LAS expanded their geographic allocation to 250-nautical mile radius, this change further improved access to pediatric organs.^12^ Finally, in March 2023 the LAS was discontinued a continuous distribution system was implemented giving each candidate a composite allocation score (CAS). This score was developed to better represent the urgency for transplant, the post-transplant outcomes, the transplant efficiency, and compensate for biologic disadvantages. Specifically, for patients on the waitlist listed at <18 years of age, 20 points are assigned. Also, candidates <12 years of age are assigned priority 1 or 2 depending on their clinical status.^13^ It is still early to determine how the change to CAS has impacted access to pediatric lung transplant candidates. An important change from the LAS is that previously, organs from donors <18 years were first offered to pediatric candidates; currently, this is not the case. The 20 extra points for pediatric access is used in lieu of prioritizing pediatric organs to pediatric candidates. Given the thoracic size difference between a child and an adult, adult donor lungs are not always appropriate for children. The impact on pediatric lung transplant outcomes is yet to be seen. CAS has indeed increased access to organs in the adult population, but it has shown little change in the pediatric access. Reassuringly in the first 18 months post implementation, the pediatric waitlist mortality has not significantly changed.^14^

Outside the United States, allocations systems vary from region to region. For example, Eurotransplant prioritizes pediatric donor lungs to pediatric candidates and cross-border agreements are designed to mitigate the scarcity of the organs. Spain uses a national priority system prioritizing pediatric patients with high clinical urgency. The United Kingdom launched the UK Lung Allocation Scheme in 2017 a 3-tier based model that prioritizes clinical urgency.^15^ Finally, both Canada and Australia are more center based regional system with no national unifying system, but pediatric patients can be given a higher priority for urgent cases^16^

There needs to be more research and advocacy to increase access to pediatric organs. Firstly, creating an allocation system that is fair to all candidates but also prioritizes children that are at a biologic disadvantage due to their size and age is key to narrowing the gap in the waitlist mortality compared to adults. More world-wide data need to be collected about the allocation systems and the impact on the pediatric waitlist trends.

Secondly, there has been slow adoption and implementation of recent the techniques and technology such as use of DCD donors, EVLP and controlled hypothermic storage to increase the donor pool for children waiting for lung transplantation. Both are underutilized in pediatric lung transplant. Worldwide, only 34 pediatric DCD transplants were done between 2004 and 2018.^17^ Some of the limiting factors for pediatric programs to adopt these techniques include the low volume of pediatric transplants and possible knowledge or education gaps as well as the high cost associated with EVLP in addition to technical considerations for a technology intended for large adult lungs. DCD donors and EVLP has the promise to increase access, but more pediatric specific experience and research is need.^11^

## ABO-incompatible pediatric lung transplantation

The shortage of suitable donor lungs, in particular, for smaller children remains a challenge. A viable strategic option in this regard is ABO-incompatible lung transplantation. The concept of ABO-incompatible solid organ transplantation is being used for elective renal (living-related) and pediatric heart transplants. In lung transplantation, anecdotal cases of unintentional ABO-incompatible transplants have been reported on. In 2012, the Toronto Group published a successful ABO-incompatible lung transplant in an infant with a blood group A, using a blood group B DCD donor, applying plasmapheresis in addition to standard immunosuppression peri-operatively.^10^ A handful more selected infants have undergone ABO-incompatible lung transplants in Hannover, Germany,^18^ and Toronto, Canada (Melinda Solomon, personal communications). ABO-incompatible lung transplants of A2 and A2B donors into blood group B and O recipients with low anti-A titers seems a viable option to explore further in the future. A Group in Japan published an intentional first living-donor lobar lung transplant (LDLLT) of a teenager receiving a single lobe from an ABO-incompatible donor and another lobe from an ABO-identical donor.^19^

## Acute, basic, and chronic lung allograft dysfunction phenotypes

Major advances have been made in the history of pediatric lung transplantation with regards to donor lung procurement and preservation, the transplant operation and surgical techniques, early post-operative management, prevention of acute cellular rejection, and prophylaxis and therapy of infectious complications. However, the overall outcome of children following lung transplantation is still inferior in comparison with other solid organ transplants.

The major hurdle for improved overall survival remains the development of chronic lung allograft dysfunction (CLAD). For the first time, the Pulmonary Council of the International Society for Heart and Lung Transplantation (ISHLT) published in 2019 a consensus document on its definition, diagnostic criteria, and approaches to therapy.^20^ In this document, CLAD is defined as a persistent decline in lung allograft function, characterized by a decrease ≥20% of forced expiratory volume in 1 s (FEV_1_) for >3 months from baseline post-transplant. All other causes of lung allograft dysfunction need to be excluded. The baseline value is calculated from the mean of the two best FEV_1_ values measured post-operatively ≥3 weeks apart, and the CLAD severity is classified based on the current FEV_1_ value compared to the baseline FEV_1_ value, i.e., CLAD 3 with a current FEV_1_ >35–50% FEV_1_ baseline value. However, this definition based on pulmonary function values is a challenge in the pediatric lung transplant population as somatic growth throughout childhood is not appropriately accounted for. Further, given the recent changes in indications for pediatric lung transplants with a relative reduction in the proportion of pediatric candidates with cystic fibrosis and a higher proportion of predominately younger children with other primary indications such as pulmonary arterial hypertension and childhood interstitial lung disease, the question arises how to correctly monitor infants and younger children for CLAD due to their inability to reliably perform spirometry.^21^ A recently published international survey of 25 pediatric lung transplant programs showed that only one-third use infant/preschool pulmonary function tests, and the vast majority of programs rely on CT chest imaging for routine surveillance and detection of suspected CLAD.^21^ Given the aforesaid diagnostic challenges in younger children, advances in technology need to be made for age-appropriate CLAD surveillance and monitoring, exploring the potential utility of other techniques such as oscillometry, currently trialed in adult lung transplant patients.^22^ To complicate the matter further, CLAD is rather considered an umbrella term as it comprises a range of phenotypes, most commonly, bronchiolitis obliterans syndrome (BOS), a small airway-centered disease process, and restrictive allograft syndrome (RAS).^23^ Patho-biologically, RAS is a rather parenchymal-pleural disease process. Thus, some authors opt to use the term endotypes instead of phenotypes of CLAD to indicate the distinctly different underlying patho-biological mechanisms of both BOS and RAS. Survival of adults developing CLAD-RAS after lung transplants is considered inferior compared to CLAD-BOS; however, data in the pediatric population is scarce to be applied for risk stratification. Thus, prospective pediatric data collection is warranted in the future.

Currently, efforts are also underway led by the ISHLT Pulmonary Professional Community to find international consensus on acute lung allograft dysfunction (ALAD) and baseline lung allograft dysfunction (BLAD), respectively. There is pediatric expert representation in both Writing Groups. The term ALAD describes a range of different clinical scenarios of multifactorial nature; ALAD is likely to be defined as a respiratory syndrome with a ≥10% FEV_1_ decline and/or worsening in oxygenation for less than 3 months, and it is distinct from primary graft dysfunction (PGD) and BLAD. However, ALAD could take place in the setting of established CLAD or as a precursor of CLAD. The concept and terminology of BLAD describes the failure of restoring normal pulmonary function for a given recipient after lung transplantation, asking the questions how frequently and why we fail to achieve this primary goal (Kieran Halloran, personal communication). To date, in the few published studies on BLAD, spirometry measurements (FEV_1_ and forced vital capacity, FVC) play a central role defining BLAD. Early outcomes of BLAD include an increased risk of severe PGD, a prolonged post-operative ventilatory support, and an extended ICU stay. The first ISHLT consensus document on BLAD focusing on definition, timing, and implications for outcomes has recently been finalized and submitted for publication (Kieran Halloran, personal communication).

As it occurs frequently in the field of pediatric lung transplantation, there are limited data to guide the application of ALAD and BLAD definitions and mechanisms in pediatrics. Further, the term ALAD is very rarely used in children to date, it is considered an umbrella term for acute graft injury. In the pediatric population, special considerations will need to be made in terms of pulmonary function measurements in the future, as spirometry is less applicable for smaller children due to the lack of test reproducibility. This also applies to any future definition of BLAD in younger children, as spirometry measurements are likely key to the BLAD diagnosis in older children and adults. Perhaps, decreased exercise tolerance or hypoxia could be useful indicators of allograft dysfunction in smaller children.

## Lung transplant outcomes: role of malnutrition, sarcopenia and frailty

As indications for lung transplantation (LTx) and patient characteristics changed over the years,^8^, ^24^ identification of major risk factors for transplant outcomes has become crucial for improving patient care and survival rates. Over the past decade, the ISHLT has analysed the role of recipient diagnosis, donor/recipient size match, and donor ischemic time, in pediatric transplantation. Although pre-transplant BMI and the level of post lung transplant functional status were captured by the International Thoracic Organ Transplant (TTX) Registry, their impact in LTx outcomes were not thoroughly studied.^25^, ^26^

Severe malnutrition in the form of low body mass index (BMI), can be considered a relative contraindication for LTx due its negative effect on graft function and overall survival.^27^ Unfortunately, nutritional assessment in patients with end-stage lung disease can be challenging; BMI calculation may not be accurate in patients with stunted growth from chronic disease, steroid use and other factors.^28^ Body composition can better estimate nutritional status and identify sarcopenia and physical frailty. There are different techniques to assess body composition^28^ but only a few have been studied in pediatric LTx patients.^29^ In addition to nutritional status, physical functional evaluation can aid clinicians to develop interventions to treat malnutrition, sarcopenia and frailty.^28^

Recent studies have looked closer to the association between functional status and patient outcomes. A single center study investigated patients’ pre-transplant health condition by capturing functional status (as degree of help with activities of daily living - ADLs), 6-minute walk test (6MWT), ambulatory status and mechanical ventilation use. Patients that were independent or needed some help with ADLs had shorter time on the ventilator. Worse outcomes with longer ICU stay were observed in patients with limited ambulation; while those patients who ambulated >500 ft in a 6MWT, had shorter ICU stay and had a trend to better 12-month survival.^30^ Other groups studied functional status captured as Lansky Play Performance Scale (LPPS) in the UNOS registry, and its association with waitlist and post-transplant outcomes. Candidates with the worst functional status, completely dependent functional status (LPPS 10–40), had a higher rate of removal from waitlist due to death or clinical deterioration. Because waitlist removal due to death or clinical deterioration occurs in 20–25% of pediatric lung transplant patients, the authors concluded that identifying at-risk candidates could guide transplant centers to develop early interventions to improve functional capacity prior to and during waitlist.^31^ Those patients with severely limited functional status (LPPS 10–40) at time of transplant had worse 1-year post-LTx outcome.^32^

Frailty is a state of decreased physical and physiological reserves, putting patients at risk of adverse health outcomes. Frail adult lung transplant candidates showed improvement in functional capacity, strength, depression and anxiety 1-year post LTx by participating in structured rehabilitation post-transplantation; they had similar outcomes compared to non-frail patients.^33^ Frailty is more difficult to identify, and it is probably underrecognized in children. Aspects of frailty, slowness, weakness, exhaustion, shrinkage, and diminished physical activity can be evaluated and scored using a modified Fried frailty assessment tool.^34^ Although reduced exercise capacity and poor functional status can be considered a contraindication for transplant, the goal of frailty assessment is not to automatically exclude very limited patients, but to identify issues and develop action plans. As more critically ill children are considered for lung transplantation,^24^ it is important to develop strategies to ameliorate malnutrition and frailty in order to optimize the functional status of pediatric lung transplant candidates, and improve outcomes.^32^

## Conclusion

Pediatric lung transplantation remains a critical life-saving therapy; however, the field is undergoing substantial changes and continues to face significant challenges that will shape its future. Evolving transplant indications, increasing patient complexity, and the growing emphasis on optimizing patient health prior to transplantation underscore the importance of a comprehensive multidisciplinary team approach. At the same time, maintaining the specialized expertise and technical skills required to care for pediatric lung transplant recipients remains challenging, as most centers perform fewer than 10 pediatric lung transplants annually and overall transplant volumes continue to gradually decline. Providers, legislators, and transplant families must work collaboratively to improve access to suitable donor organs and advocate for advances in pediatric-specific research, technology, and innovation. In parallel, the pediatric lung transplant community should continue to strengthen global collaboration through the sharing of expertise, data, and clinical experience, thereby advancing the field and supporting the education and development of future generations of pediatric lung transplant providers.

## Financial disclosure

No funding.

## Data availability statement

No original data included.

## Declaration of Competing Interest

The authors declare that they have no known competing financial interests or personal relationships that could have appeared to influence the work reported in this paper.
